# The Role of Central Command in the Increase in Muscle Sympathetic Nerve Activity to Contracting Muscle During High Intensity Isometric Exercise

**DOI:** 10.3389/fnins.2021.770072

**Published:** 2021-12-02

**Authors:** Daniel Boulton, Chloe E. Taylor, Simon Green, Vaughan G. Macefield

**Affiliations:** ^1^School of Science and Health, Western Sydney University, Sydney, NSW, Australia; ^2^Neuroscience Research Australia, Sydney, NSW, Australia; ^3^School of Medicine, Western Sydney University, Sydney, NSW, Australia; ^4^Baker Heart and Diabetes Institute, Melbourne, VIC, Australia

**Keywords:** metaboreflex, muscle contraction, muscle sympathetic nerve activity, central command, pressor response

## Abstract

We previously demonstrated that muscle sympathetic nerve activity (MSNA) increases to contracting muscle as well as to non-contracting muscle, but this was only assessed during isometric exercise at ∼10% of maximum voluntary contraction (MVC). Given that high-intensity isometric contractions will release more metabolites, we tested the hypothesis that the metaboreflex is expressed in the contracting muscle during high-intensity but not low-intensity exercise. MSNA was recorded continuously via a tungsten microelectrode inserted percutaneously into the right common peroneal nerve in 12 participants, performing isometric dorsiflexion of the right ankle at 10, 20, 30, 40, and 50% MVC for 2 min. Contractions were immediately followed by 6 min of post-exercise ischemia (PEI); 6 min of recovery separated contractions. Cross-correlation analysis was performed between the negative-going sympathetic spikes of the raw neurogram and the ECG. MSNA increased as contraction intensity increased, reaching mean values (± SD) of 207 ± 210 spikes/min at 10% MVC (*P* = 0.04), 270 ± 189 spikes/min at 20% MVC (*P* < 0.01), 538 ± 329 spikes/min at 30% MVC (*P* < 0.01), 816 ± 551 spikes/min at 40% MVC (*P* < 0.01), and 1,097 ± 782 spikes/min at 50% MVC (*P* < 0.01). Mean arterial pressure also increased in an intensity-dependent manner from 76 ± 3 mmHg at rest to 90 ± 6 mmHg (*P* < 0.01) during contractions of 50% MVC. At all contraction intensities, blood pressure remained elevated during PEI, but MSNA returned to pre-contraction levels, indicating that the metaboreflex does not contribute to the increase in MSNA to contracting muscle even at these high contraction intensities.

## New and Noteworthy

We recorded muscle sympathetic nerve activity (MSNA) to contracting leg muscles at intensities up to 50% of maximum, followed by 6 min of post-exercise ischemia (PEI). MSNA and mean arterial pressure (MAP) increased incrementally at each contraction intensity but MSNA returned to baseline levels at the end of exercise despite MAP remaining elevated. We conclude that the increase in MSNA to contracting muscle is primarily due to the increase in central drive, not the metaboreflex.

## Introduction

The cardiovascular demands of exercising skeletal muscle require close regulation by neural mechanisms to increase cardiac output, blood pressure and to redistribute blood flow to contracting muscle. During exercise muscle sympathetic nerve activity (MSNA), which is vasoconstrictor in function, is governed by several mechanisms, including a centrally mediated efferent drive (central command), resetting of the arterial baroreflex and reflexes generated by stimulation of mechanically and metabolically-sensitive group III and IV afferent endings in the contracting muscle. When blood flow is reduced during isometric exercise metabolic by-products, such as hydrogen ions, lactate and nitric oxide (Victor et al., 1988; Dinenno and Joyner, 2003), accumulate over time and stimulate the muscle metaboreflex, which leads to an increase in MSNA and blood pressure that persists during the post-exercise ischemia (PEI) induced by occlusion of the arterial supply.

While metaboreceptor-mediated increases in MSNA to non-contracting skeletal muscle during isometric exercise have been well documented since the first demonstration in humans (Alam and Smirk, 1937), the role of this mechanism in the control of MSNA to the *contracting* muscle is less well understood. For instance, MSNA to contracting muscle has been reported to decrease during isometric dorsiflexion of the ankle (Wallin et al., 1992), but in another study it did not change during dorsiflexion of the toes (Hansen et al., 1994). Although the reason for these disparate results is not entirely clear, methodological and analytical differences may contribute to some extent. Moreover, neither of these studies specifically addressed whether the metaboreflex acts on contracting muscle.

Since evidence of the muscle metaboreflex has only been defined by observations in non-contracting muscle, we recently recorded *MSNA to contracting leg muscles* during low-intensity isometric dorsiflexion of the ankle, before and during 2 min of PEI. MSNA increased for the duration of the 2-min contraction but promptly returned to baseline during 2 min of PEI, suggesting that the muscle metaboreflex was not involved in the sympathoexcitation to the contracting muscles (Boulton et al., 2014). In a subsequent study, we used a more sensitive means of measuring MSNA to contracting muscle: using spike-discrimination software to separate the spikes generated by sympathetic axons from those generated by muscle spindle and Golgi tendon organ afferents and motor axons, and the interference produced by electromyographic (EMG) activity. Using this approach we showed that short periods (1 min) of isometric ankle dorsiflexion increased MSNA to contracting muscle after 15 s, but that electrically evoked contractions did not, and MSNA returned to baseline as soon as the contraction ceased (Boulton et al., 2016). Given that electrically evoked contractions bypass central command this would suggest that central command is responsible for the increase in MSNA to contracting muscle, though this may only true for the low-intensity (∼10% of maximum) voluntary contractions performed in that study.

More recently, we used a longer period of contraction (4 min) and a longer period of PEI (6 min) to assess the time-course of changes in MSNA to the contracting muscles. Although MSNA increased to both the contracting (ipsilateral) and non-contracting (contralateral) muscles during these low-intensity isometric ankle dorsiflexions (10% of maximum), during the period of PEI MSNA only remained elevated in the non-contracting muscle; conversely, MSNA to the contracting muscle promptly returned to and remained at baseline levels for the duration of PEI (Boulton et al., 2018). Moreover, we observed an augmented increase in MSNA to the non-contracting muscles when the contractions were performed in the presence of ongoing ischemia, which we reasoned would augment metaboreceptor drive, but MSNA to the contracting muscles nevertheless returned to baseline levels. What is not known is whether the increase in MSNA to contracting muscle, and the absence of maintained MSNA during PEI, holds true during high-intensity contractions: does MSNA increase with contraction intensity and is the metaboreflex expressed ipsilaterally following stronger isometric contractions? The purpose of the current study was to test the hypothesis (i) that MSNA to the contracting muscle increases in an intensity-dependent manner, and (ii) that the metaboreflex is expressed to the contracting limb following high intensity contractions, owing to the greater accumulation of metabolites.

## Materials and Methods

### Participants and Ethics

This study was conducted in accordance with the Declaration of Helsinki and with the approval of the Human Research Ethics Committee of Western Sydney University (H8728). Twelve healthy participants (seven males, five females) aged 20–57 years (mean age 31 ± 13 (SD) years) participated in the study and provided informed written consent. Participants were instructed to abstain from consumption of stimulants or depressants (for example, caffeine and alcohol) and vigorous exercise 24 h prior to the study. Individuals who smoked, took regular medication or suffered from neurological or cardiorespiratory illnesses were excluded from participating in the study.

### Recording Procedures

Participants were positioned semi-recumbent in a chair with their torso at ∼45°, legs supported horizontally, and feet strapped in a slightly plantarflexed position (95°) to a footplate. After locating the course of the common peroneal nerve at the fibular head by electrical stimulation (Stimulus Isolator, ADInstruments, Sydney, Australia) through a 2 mm diameter probe (0.2 ms pulse at 1 Hz, 2–10 mA current), a tungsten microelectrode (Frederick Haer and Co., Bowdoinham, ME, United States) was inserted into the skin overlying the nerve. A reference microelectrode with an uninsulated tip was inserted subcutaneously approximately 1 cm from the active microelectrode. Further stimulation was used at a lower current (≤ 1 mA) until twitches of the ankle or toe dorsiflexor muscles could be detected at 20 μA, without radiating paraesthesia. To verify that the fascicle supplied muscle, the following additional observations were made prior to beginning the protocol: an increase in the rate of firing of positive-going muscle spindle afferents upon passive stretch or palpation of the parent muscle or tendon, but not light stroking of the skin, and a sustained increase of spontaneous cardiac-locked bursts of muscle sympathetic nerve activity for the duration of a maximal inspiratory apnea. The microelectrode tip was then manually guided within the fascicle until oligounitary bursts of MSNA could be detected. Neural activity was amplified (gain 2 × 10^4^) and filtered (bandpass 0.3–5.0 kHz) using an isolated amplifier and headstage (NeuroAmpEX, ADInstruments, Sydney, Australia) and stored on computer (10 kHz sampling) using a computer-based data acquisition and analysis system (PowerLab 16–35 hardware and LabChart 7 software; ADInstruments, Sydney, Australia).

A single lead (II) electrocardiogram (0.3–1 kHz) was recorded with Ag-AgCl surface electrodes (BioAmp, PowerLab, ADInstruments) on the chest and sampled at 2 kHz (bandpass 0.3–1 kHz). Respiration (DC-100 Hz) was recorded using a strain gauge transducer (Pneumotrace II; UFI, Morro Bay, CA, United States) around the chest and sampled at 100 Hz. Continuous, non-invasive beat-to-beat blood pressure was measured at 400 Hz from the middle finger of the right hand using digital arterial plethysmography (Finometer Pro, Finapres Medical Systems, Enchede, Netherlands). Electromyographic (EMG) activity was recorded with Ag-AgCl surface electrodes over the proximal and distal belly of tibialis anterior and was sampled at 2 kHz (10 Hz–1 kHz). Force was measured using a load cell (Aluminum S Type EG PT) connected to a footplate, amplified (gain × 200, bandpass DC-10 Hz; Quad Bridge Amplifier, ADInstruments, Sydney, Australia), sampled at 100 Hz and normalized to the MVC of the participant. To control blood flow to the right leg, an 18 cm sphygmomanometer cuff was wrapped around the right upper thigh and attached to a rapid cuff inflation system (AG101 and E20; Hokanson, Bellevue, WA, United States), which was set to a suprasystolic pressure (200 mmHg). Ischemia was verified by the absence of a pulse in an ipsilateral toe using a piezoelectric pulse plethysmograph (UFI, Morro Bay, CA, United States).

### Experimental Protocol

Before beginning the exercise protocol, the maximal voluntary contraction (MVC) force of the right ankle dorsiflexors was determined from at least two 3-s attempts. A 5-min baseline period was recorded once a stable MSNA recording had been established. Participants performed a series of 2-min sustained isometric dorsiflexion contractions followed immediately by 6 min of muscle ischemia of the previously contracting leg and at least 6 min of rest to allow all measurements to return to baseline levels. Dorsiflexions were performed in ascending 10% increments from 10 to 50%MVC because of the increasing risk of losing the recording site with higher intensity contractions. Participants were instructed to slowly increase force to the target level so as to ensure stability of the microelectrode recording. During the strongest contractions (40 and 50% MVC) the recording site was lost for four participants, such that the entire protocol was completed for eight of the 12 experiments. Participants were asked to report their perceived level of exertion on the Borg (6–20) scale at the end of each minute of contraction and their level of pain or discomfort on a pain scale (0–10) every 2 min during ischemia, providing three time points for each period of ischemia. To test the effect of ischemia alone, an additional 1-min period of muscle ischemia was recorded after all measurements had returned to baseline levels and at least 3 min prior to the next contraction. Participants were also asked to provide a score on the pain scale during the periods of ischemic rest.

### Muscle Sympathetic Nerve Activity Analysis

Analysis of MSNA involved extracting negative-going sympathetic spikes from the raw neurogram to avoid inclusion of spikes generated by positive-going myelinated axons, such as muscle spindle or Golgi tendon afferents, motor axons and EMG infiltrating the neurogram. Window discriminator software (Spike Histogram, LabChart 2.5, ADInstruments, Sydney, Australia) was used to detect negative-going (half width 0.2–0.6 ms) sympathetic spikes. The ECG was shifted back in time (1.1–1.3 s) to account for the delay due to the slow conduction of sympathetic nerve activity (Fagius and Wallin, 1980). The time of occurrence of the R-waves in the ECG were computed with appropriately wide time-windows to reflect the longer duration of the events. As described previously (Bent et al., 2006; Fatouleh and Macefield, 2013; Boulton et al., 2016), autocorrelation histograms for ECG signals and cross-correlation and post-stimulus time histograms between MSNA and ECG, were generated by the Spike Histogram software (50 ms bins). Discriminator parameters for MSNA were set to ensure that robust cardiac modulation of spike counts was apparent in the cross-correlation histograms. MSNA spike counts were measured in 2-min epochs. MSNA spike counts were collected from 600 ms periods after each R-wave (i.e., diastole), which also centered about a peak spike count in the post-stimulus time histogram. Any contraction intensities where MSNA bursts could not be detected visually or by sound during PEI or recovery were omitted from the analysis and the contraction was repeated at the same intensity after sufficient rest and after acquiring a new recording site.

A two-way repeated measures ANOVA, coupled with Tukey’s test for multiple comparisons (Prism 5.0, GraphPad Software Inc., San Diego, CA, United States) was performed to test for the main effects and interactions between “time” and “intensity” (10–50%MVC). Significance was set at *P* < 0.05 and results are expressed as mean ± SD (SE in figures).

## Results

**FIGURE 1:**
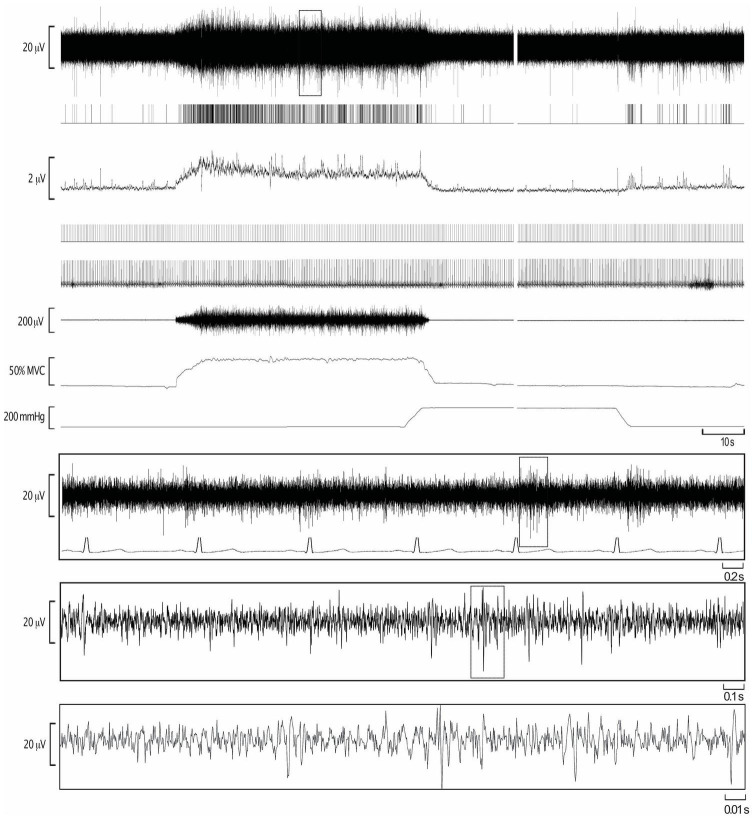
Experimental records from one participant during isometric dorsiflexion of the ankle at 50% of maximal voluntary contraction (MVC), followed by post-exercise ischemia (PEI) and rest. The extracted negative-going sympathetic spikes are shown as standard pulses (MSNA), together with standard pulses indicating the R-waves of the ECG. These event markers were used to construct the autocorrelation histogram for the ECG and the cross-correlation histogram between MSNA and ECG.

**FIGURE 2:**
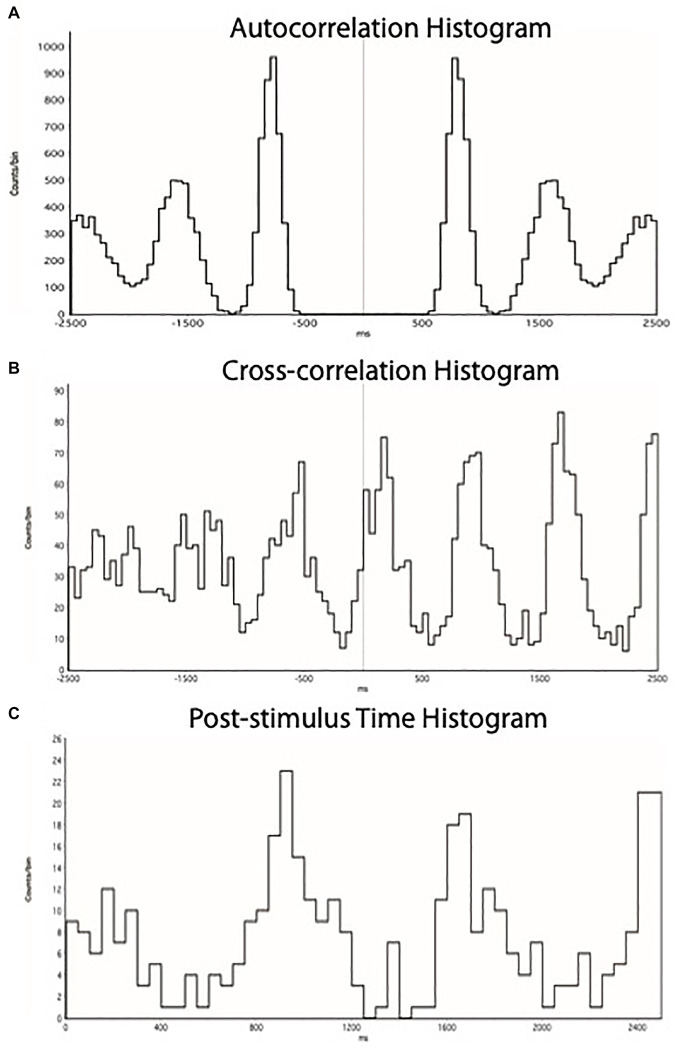
**(A)** ECG autocorrelation histogram, showing times of occurrence of 5 cycles of R-waves. **(B)** Cross-correlation histogram between MSNA and ECG, showing times of occurrence of sympathetic spikes as a function of the 5 cycles of R-waves shown in **(A)**. **(C)** Post-stimulus time histogram, showing the times of occurrence of sympathetic spikes relative to the triggering R-wave. Each set of histograms was generated for each specific epoch of the recording to discriminate cardiac-locked negative-going sympathetic spikes from the raw neurogram.

All 12 participants (seven males, five females) successfully completed a series of sustained isometric ankle dorsiflexions at 10, 20, and 30% MVC while MSNA was recorded to the contracting muscle. Eight of these participants (four males, four females) also completed contractions at 40 and 50%MVC, while the neural recording was lost in the four participants who did not complete the higher intensity contractions. Experimental records from one participant during a 2-min contraction at 50%MVC, followed by 6 min of post-exercise ischemia (PEI), are shown in Figure 1. MSNA data from the baseline and inter-trial rest periods are presented in Table 1. Measured from the post-stimulus time histogram (Figure 2), the number of sympathetic spikes during the initial 5-min baseline period (76 ± 35 spikes/min, *P* > 0.45) was similar to that recorded during the rest period before each contraction, as measured throughout the protocol (75 ± 39 spikes/min, *P* = 0.45), as well as during the recovery period after PEI (77 ± 53 spikes/min, *P* > 0.78). Muscle ischemia on its own, applied during the rest periods, had no effect on MSNA (71 ± 26 spikes/min, *P* = 0.5) when compared to the baseline period.

**TABLE 1:** Sympathetic spike counts at rest, during the 2 min of exercise, during the 6 min of post-exercise ischemia (PEI), split into 2-min epochs (PEI-1, PEI-2, and PEI-3) and during recovery.

Intensity (%MVC)	Rest	Exercise	PEI-1	PEI-2	PEI-3	Recovery
**10**	73 ± 26	207 ± 210*	85 ± 103	76 ± 72	68 ± 71	71 ± 37
**20**	77 ± 36	270 ± 189*	71 ± 45	56 ± 39	67 ± 55	64 ± 42
**30**	71 ± 32	538 ± 329*	76 ± 50	60 ± 51	60 ± 46	78 ± 53
**40**	81 ± 53	816 ± 551*	101 ± 94	88 ± 94	83 ± 92	79 ± 63
**50**	76 ± 54	1,097 ± 782*	100 ± 92	74 ± 62	76 ± 54	102 ± 72

Significant increases are indicated with an asterisk (*P < 0.05).

**FIGURE 3:**
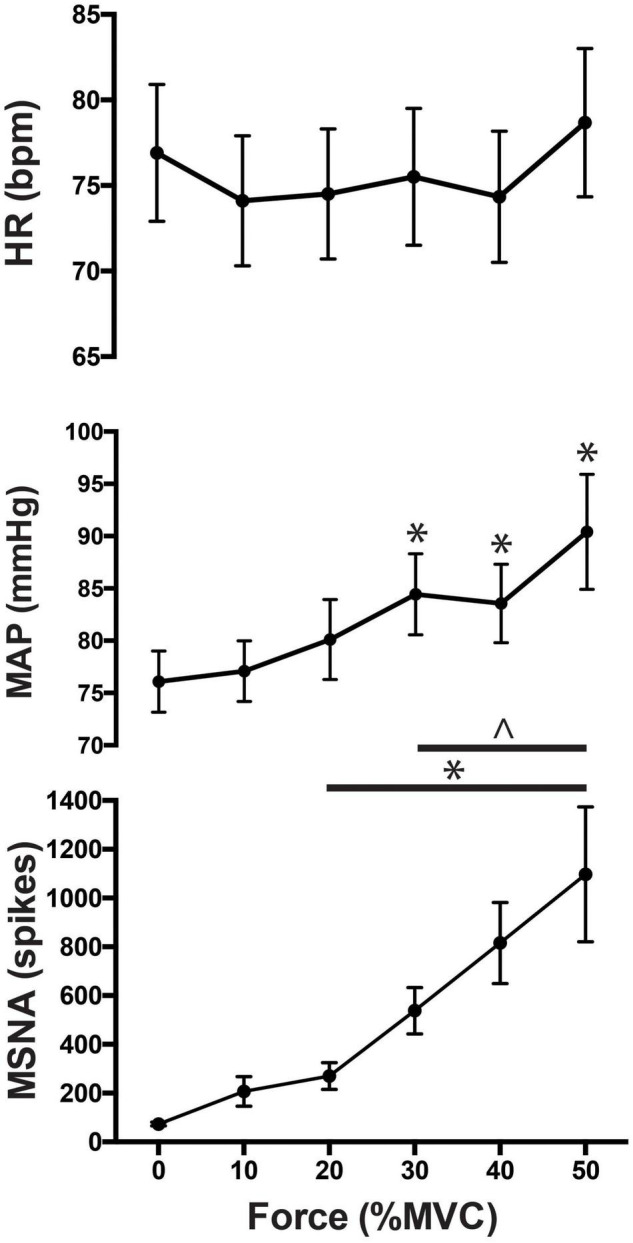
MSNA (spikes/min), normalized mean arterial pressure (MAP) and heart rate responses to contractions at 0 (baseline), 10, 20, 30, 40, and 50% of maximal voluntary contraction (MVC). Main effects of time are indicated with an asterisk (*P < 0.05). Significant interactions are represented by ∧ (*P* < 0.05).

**FIGURE 4:**
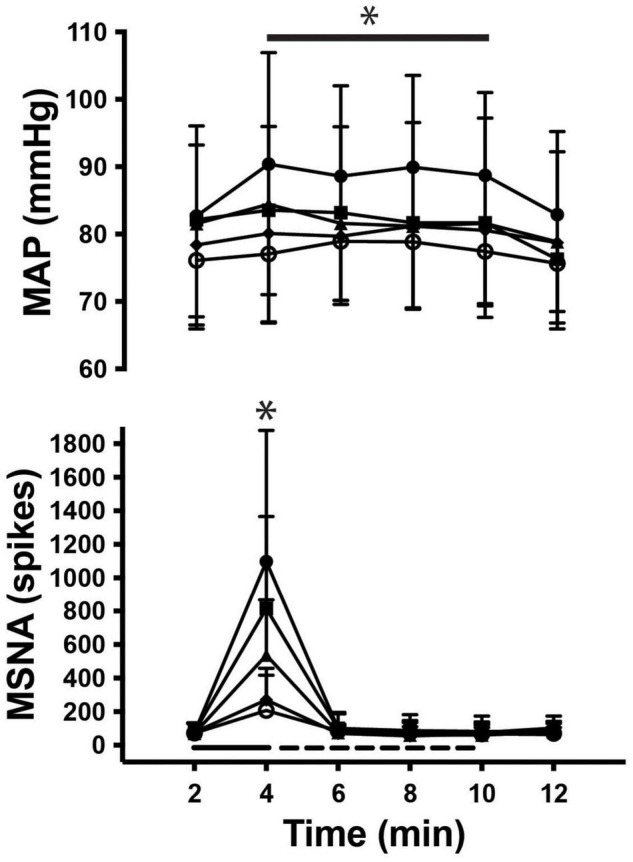
Time course of MSNA and mean arterial pressure (MAP) during rest, contraction (**__**), post-exercise ischemia (**- - -**) and recovery. Main effects of time are indicated with an asterisk (*P < 0.05).

As illustrated in Figure 3, there was a significant main effect of time on MSNA [*F*_(5, 308)_ = 83.7; *P* < 0.001] during contractions, as well as a main effect of intensity [*F*_(4, 308)_ = 4.1; *P* < 0.01] and a significant interaction [*F*_(20, 308)_ = 8.0; *P* < 0.001]. MSNA increased further as the intensity of contraction increased, peaking at 1,511 ± 994% (1,097 ± 782 spikes/min; *P* < 0.01) above rest during contractions at 50%MVC. There were also significant increases in MSNA from pre-contraction to contraction intensities of 10% (207 ± 210 spikes/min, *P* = 0.04), 20% (270 ± 189 spikes/min, *P* < 0.01), 30% (538 ± 329 spikes/min, *P* < 0.01), and 40% of MVC (816 ± 551 spikes/min, *P* < 0.01). The increase in MSNA during 40 and 50%MVC was significantly greater compared to contractions at the lower intensities (*P* < 0.03). However, at all intensities of contraction MSNA returned to pre-contraction levels during PEI (76 ± 69 spikes, *P* > 0.2 compared to baseline) and remained at this level for the remaining period of rest (Figure 4).

As shown in Figure 3 mean arterial pressure increased in an intensity-dependent manner during exercise, peaking 18 ± 10% above baseline during 50% MVC (76 ± 3 mmHg vs. 90 ± 6 mmHg; *P* = 0.01). Heart rate did not significantly increase from rest during exercise or PEI, only increasing by more than a few beats per minute during contractions at 50%MVC (rest v exercise: 73 ± 14 vs. 79 ± 13 bpm; *P* < 0.05). MSNA remained elevated for the duration of the contraction (*P* < 0.05), as shown in Figure 4. Two-way ANOVA demonstrated that there was a significant main effect of contraction intensity on normalized mean arterial pressure [*F*_(4, 308)_ = 28.52; *P* < 0.0001], a significant effect of time [*F*_(5, 308_) = 3.528; *P* < 0.01], but no significant interaction [*F*_(20, 308)_ = 0.479; *P* = 0.97].

Table 2 presents grouped ratings of perceived exertion during contractions and perceived discomfort during PEI. Ratings of perceived exertion at higher intensities (30–50% MVC) were significantly greater than perceived exertion at 10% MVC [*F*_(4, 380)_ = 9.1; *P* < 0.01], being 8 ± 2 during contractions at 10% yet 14 ± 3 at 50% MVC. Perceived discomfort during PEI was 3.5 ± 0.3 out of 10 and did not change throughout the periods of PEI (*P* = 0.53).

**TABLE 2:** Participants’ rating of perceived exertion (Borg scale = 6–20) during each contraction and rating of discomfort (0–10) during post-exercise ischemia (PEI), or during ischemia alone.

	Interval	10%	20%	30%	40%	50%	Ischemia only
**Perceived exertion**	1	8 ± 2	10 ± 2	11 ± 3*	13 ± 3*	14 ± 3*	
	2	8 ± 2	10 ± 2	12 ± 3*	13 ± 3*	14 ± 3*	
**Perceived discomfort**	1	3 ± 1	4 ± 1	3 ± 1	3 ± 1	4 ± 1	3 ± 1
	2	3 ± 1	4 ± 1	3 ± 1	4 ± 1	4 ± 1	
	3	3 ± 1	4 ± 1	3 ± 1	4 ± 1	4 ± 1	

Values are presented as mean ± SD at each 1-min interval during the contraction, and during each 2-min interval during PEI. Higher scores indicate more effort or discomfort. Significant changes from 10%MVC are indicated with an asterisk (*P < 0.05).

## Discussion

We have shown that MSNA increases to the contracting muscle in an intensity-dependent manner during isometric contractions of the ankle dorsiflexors up to intensities of 50% of MVC, the highest we attempted, and remained elevated for the entire 2 min of contraction. Never before has MSNA been able to be recorded during such strong contractions, owing to the infiltration of the nerve signal from neural spikes generated by muscle afferents and motor axons and from the nerve signal being swamped by EMG from the contracting muscles. Moreover, we found no evidence that accumulation of metabolites during the isometric contraction and during the 6 min of PEI is responsible for this increase in MSNA, despite the greater release and accumulation of metabolites during exercise at these higher intensities. MSNA was augmented throughout each contraction, increasing as force and perceived exertion increased, but returned to pre-contraction levels when the contraction ceased.

Evidently, the fact that mean arterial pressure remained elevated during the 6 min of PEI can be explained by the well-known increase in sympathetic vasoconstrictor drive to non-contracting muscle, i.e., the metaboreflex. That perceived exertion, as measured using the Borg scale, increased with contraction intensity supports the obvious conclusion that central command increased with increasing contraction intensity. Indeed, during contractions at 50%MVC perceived exertion reached a Borg rating of 14.3 ± 3.3, corresponding to the description of the task as being “hard.” These findings support our first hypothesis that MSNA increases in an essentially linear fashion with contraction intensity. What we do not know is whether the accumulation of metabolites interacted with this centrally generated increase in MSNA to the contracting muscle, but the fact that MSNA rapidly returned to baseline levels at the end of each contraction, despite the ongoing stimulation of metaboreceptors in the previously contracted muscle during the 6 min of PEI, would support our conclusion that the metaboreflex is not expressed in the contracting muscle. Accordingly, on the basis of these data, we reject our second hypothesis—that the muscle metaboreflex increases sympathetic vasoconstrictor drive to contracting muscle during sustained high-intensity isometric contractions.

What is responsible for the increase in MSNA to contracting muscle? The feed-forward mechanism of central command is a plausible mechanism: a prompt increase in MSNA to contracting muscle could counteract the rapid vasodilatation and rise in muscle blood flow at the onset of exercise (Tschakovsky et al., 2004; Reeder and Green, 2012) and subsequent fall in arterial pressure (Sprangers et al., 1991). The latter would produce a sympathetically mediated vasoconstrictor response by unloading of the baroreceptors, which would prevent hypotension and contribute to the pressor response to exercise (Masuki and Nose, 2003). In our previous studies, MSNA increased within the first 30 s of contraction and returned to pre-contraction levels even when PEI was imposed (Boulton et al., 2014, 2018). The role of central command in the control of MSNA has also been demonstrated during intermittent and dynamic exercise, where the increases in MSNA to non-contracting muscle coincides with the centrally generated increases in EMG and heart rate (Victor et al., 1989). Indirect evidence of central command in the control of MSNA was also presented by Matsukawa et al. (2012) when they demonstrated a direct and rapid effect of central command on sympathetic outflow to the coronary vascular bed. Moreover, we recently showed that MSNA increases to contracting muscle more than to non-contracting muscle during weak isotonic contractions, again supporting the idea that central command is a major determinant of the increase in MSNA to contracting muscle (Taylor et al., 2019).

Although central command appears to be an important mechanism in the control of MSNA to contracting muscle, it is possible that a stronger exercise stimulus is required to evoke the muscle metaboreflex in the active muscle. Previous attempts to activate the metaboreflex in the contracting muscle have failed, despite being expressed in the contralateral non-contracting muscle during low-intensity prolonged isometric contractions (Boulton et al., 2018). Activation of the metaboreflex would have been more pronounced in the present study because of the stronger and longer contractions, creating an environment where the deprivation of oxygen delivery to active skeletal muscle was even greater than achieved during rhythmic handgrip exercise (Ichinose et al., 2011), for example. However, even after 2 min of exercise at ∼50% MVC, MSNA returned to pre-contraction levels for the duration of PEI. As noted above, the metaboreflex was clearly activated because arterial pressure was augmented for the duration of PEI, while heart rate returned to resting levels following the end of each contraction.

While these findings strengthen the argument that MSNA to contracting muscle is governed more by central command than the metaboreflex, we must acknowledge the potential contribution of the mechanoreflex to the contraction-induced increase in MSNA. We had previously shown that electrically evoked contractions of the tibialis anterior muscle, sufficient to produce an ankle dorsiflexion force of 10% MVC, did not increase MSNA to the contracting muscle, whereas a voluntary contraction of equal intensity did (Boulton et al., 2016). However, these studies were limited in that they only used brief (1 min) contractions of low intensity to avoid metaboreflex activation. We had tried to electrically stimulate the muscle at intensities sufficient to generate higher forces, but this was difficult: we used an intramuscular tungsten microelectrode inserted into one or more motor-points of the muscle to evoke contractions of tibialis anterior, and needed to limit the stimulation frequency to 20 Hz to (i) keep this within the physiological range of maximal motor unit firing rates (Macefield et al., 2000) and (ii) provide enough time within the interstimulus intervals to count the sympathetic spikes. Of course, it may be that the mechanoreflex does contribute importantly at the higher contraction intensities used in the current study, and this will remain unknown until we can achieve electrically evoked contractions of comparable intensities.

Actually, we do not know much about the contribution of the mechanoreflex in humans, other than that mechanical stimulation induced by brief stretch applied to the ankle plantarflexor muscles has been reported to evoke a rapid (within 5 s) but transient increase in MSNA to the *contralateral* dorsiflexors (Cui et al., 2006). What has *not* been done is to record MSNA during stretch of the ipsilateral muscle to which MSNA is being recorded, no doubt because of the risks of dislodging the microelectrode. Nevertheless, if we assume that the effects of mechanoreflex stimulation are transient, it would be difficult to explain the sustained increase in MSNA during the 2-min volitionally generated contractions at increasing intensities shown in the current study. Moreover, passive stretch of muscle by no means emulates what goes on during a voluntary contraction: in parallel with the increase in alpha motoneurone activity to the skeletal muscle there is an increase in gamma motoneurone (fusimotor) drive to the muscle spindles—which are sensitive to length changes within the muscle—that prevents tonically active muscle spindles from being unloaded during the extrafusal contraction and recruits silent muscle spindle afferents (Macefield and Knellwolf, 2018). This causes a net increase in excitatory drive to the alpha motoneurone pool through the spinal stretch reflex. In addition, the increase in force causes recruitment of Golgi tendon organ afferents which, being located in series with motor units, are sensitive to force but insensitive to passive stretch when the muscle is relaxed (Macefield and Knellwolf, 2018). Importantly, there is no evidence that passive muscle stretch actually increases the firing of group III muscle afferents in humans; only one study has recorded from these high-threshold mechanoreceptors in humans and none of them were sensitive to muscle stretch (Simone et al., 1994). We are left with the conclusion that passive muscle stretch is a very poor model of involuntary contraction.

What is responsible for the increase in MSNA during a contraction? Using functional magnetic resonance imaging (fMRI), we previously documented changes in cortical and subcortical activity during 2 min of isometric handgrip exercise (at 40% of maximum) and 6 min of PEI (Sander et al., 2010). Increases in BOLD (Blood Oxygen Level Dependent) signal intensity occurred in the contralateral primary motor cortex and cerebellum during the contraction, matching the effort profile and ceasing at the conclusion of the contraction. Conversely, during PEI there were progressive increases in signal intensity in the contralateral insula and primary and secondary somatosensory cortices that were sustained during the period of PEI; there were also progressive decreases in the perigenual anterior cingulate and midcingulate cortices (Sander et al., 2010). Within the brainstem there was a progressive activation of the medial and lateral dorsal medulla, corresponding to the nucleus tracts solitarius (NTS) and rostral ventrolateral medulla (RVLM), respectively, during the contraction phase that was sustained during the period of PEI. It is known that metaboreceptor afferents project to the NTS and thence onto the RVLM—the primary output nucleus for MSNA—but these changes likely reflect the progressive increase in MSNA to *non-contracting* muscle that is sustained during PEI, not the pattern we see to contracting muscle, in which MSNA returns toward control levels during the period of PEI. As for the cortical and cerebellar changes, these likely reflect the increase in central command as well as the sensory and affective components of the maneuver (Sander et al., 2010).

### Methodological Considerations

Recording MSNA from a contracting limb presents challenges that do not occur in the more typical microneurographic experiments, where MSNA is recorded from a resting limb. Minimizing the degree of movement that occurs in the immediate vicinity of the microelectrodes is a major challenge as even minute movements could cause the microelectrode to be dislodged from the fascicle from which MSNA was being recorded at rest. To minimize movement of the microelectrode, we provided specific instructions on how to perform the dorsiflexion contraction: we asked participants to gradually increase force to the target level over 5–10 s, to visualize isolating the tibialis anterior muscle and not to pull or push from the hip or thigh of either leg. The protocol did not progress until the participant had mastered the movement through low intensity (<5%MVC) practice. The slowly ramping contraction to the target force may well explain why heart rate did not increase in an intensity-dependent manner: heart rate tends to fall when attending to a task. Additionally, the sphygmomanometer cuff was smaller (18 cm) than that the 22 cm cuff used in our previous studies (Boulton et al., 2014, 2016, 2018) and was inflated gradually over the final 10 s of contraction. This meant the displaced tissue during cuff inflation was more proximal on the thigh and away from the recording site, providing us with more successful recordings due to these adjustments. Despite our best efforts, the nerve recording could not be maintained during contractions at 40 and 50%MVC for four participants. We have used cross-correlation analysis to extract the number of sympathetic spikes in the neurogram to circumvent the infiltration of other neural (e.g., motor axons, muscle spindle and Golgi tendon organ afferents) and non-neural (e.g., EMG) activity. This approach has been used effectively to assess MSNA to contracting muscle in our previous studies in which contraction intensities were lower (Boulton et al., 2016, 2018), and has allowed us to quantify sympathetic outflow to contracting muscle at intensities of up to 50% of maximum.

## Conclusion

This study builds on our recent observations of increases in MSNA to contracting skeletal muscle during mild voluntary contractions and illustrates that sympathetic outflow to contracting skeletal muscle continues to increase as contraction intensity increases. This robust increase in MSNA to contracting muscle is not maintained during PEI where the muscle metaboreflex has been elicited, providing further evidence that MSNA to contracting muscle is controlled primarily by central command and not the muscle metaboreflex.

## Data Availability Statement

The raw data supporting the conclusions of this article will be made available by the authors, without undue reservation.

## Ethics Statement

The studies involving human participants were reviewed and approved by the Western Sydney University Human Research Ethics Committee. The patients/participants provided their written informed consent to participate in this study.

## Author Contributions

DB, CT, SG, and VM conceived the study. DB and CT acquired the data. DB analyzed the data and drafted the manuscript. CT, SG, and VM contributed significantly to the interpretation of the data and to the final version of the manuscript. All authors have approved the final version of the manuscript and agreed to be accountable for all aspects of the work.

## Conflict of Interest

The authors declare that the research was conducted in the absence of any commercial or financial relationships that could be construed as a potential conflict of interest.

## Publisher’s Note

All claims expressed in this article are solely those of the authors and do not necessarily represent those of their affiliated organizations, or those of the publisher, the editors and the reviewers. Any product that may be evaluated in this article, or claim that may be made by its manufacturer, is not guaranteed or endorsed by the publisher.

## References

[B1] Alam M. Smirk F. H. (1937). Observations in man upon a blood pressure rasing reflex arising from the voluntary muscles. *J. Physiol.* 89 372–383. 10.1113/jphysiol.1937.sp003485 16994867 PMC1395054

[B2] Bent L. R. Bolton P. S. Macefield V. G. (2006). Modulation of muscle sympathetic bursts by sinusoidal galvanic vestibular stimulation in human subjects. *Exp. Brain Res.* 174 701–711. 16721608 10.1007/s00221-006-0515-6

[B3] Boulton D. Taylor C. E. Green S. Macefield V. G. (2018). The metaboreflex does not contribute to the increase in muscle sympathetic nerve activity to contracting muscle during static exercise in humans. *J. Physiol.* 596 1091–1102. 29315576 10.1113/JP275526 PMC5851889

[B4] Boulton D. Taylor C. E. Macefield V. G. Green S. (2014). Effect of contraction intensity on sympathetic nerve activity to active human skeletal muscle. *Front. Physiol.* 5:194. 10.3389/fphys.2014.00194 24917823 PMC4042086

[B5] Boulton D. Taylor C. E. Macefield V. G. Green S. (2016). Contributions of central command and muscle feedback to sympathetic nerve activity in contracting human skeletal muscle. *Front. Physiol.* 7:163. 10.3389/fphys.2016.00163 27242537 PMC4865629

[B6] Cui J. Blaha C. Moradkhan R. Gray K. S. Sinoway L. I. (2006). Muscle sympathetic nerve activity responses to dynamic passive muscle stretch in humans. *J. Physiol.* 576 625–634. 10.1113/jphysiol.2006.116640 16873399 PMC1890351

[B7] Dinenno F. A. Joyner M. J. (2003). Blunted sympathetic vasoconstriction in contracting skeletal muscle of healthy humans, Is nitric oxide obligatory? *J. Physiol.* 553 281–292. 10.1113/jphysiol.2003.049940 12949223 PMC2343482

[B8] Fagius J. Wallin B. G. (1980). Sympathetic reflex latencies and conduction velocities in normal man. *J. Neurol. Sci.* 47 433–448. 10.1016/0022-510x(80)90098-2 7420119

[B9] Fatouleh R. Macefield V. G. (2013). Cardiorespiratory coupling of sympathetic outflow inhumans, a comparison of respiratory and cardiacmodulation of sympathetic nerve activityto skin and muscle. *Exp. Physiol.* 98 1327–1336. 10.1113/expphysiol.2013.072421 23625953

[B10] Hansen J. Thomas G. D. Jacobsen T. N. Victor R. G. (1994). Muscle metaboreflex triggers parallel sympathetic activation in exercising and resting human skeletal muscle. *Am. J. Physiol. Heart Circ. Physiol.* 266 H2508–H2514. 10.1152/ajpheart.1994.266.6.H2508 8024012

[B11] Ichinose M. Delliaux S. Watanabe K. Fujii N. Nishiyasu T. (2011). Evaluation of muscle metaboreflex function through graded reduction in forearm blood flow during rhythmic handgrip exercise in humans. *Am. J. Physiol. Heart Circ. Physiol.* 301 H609–H616. 10.1152/ajpheart.00076.2011 21602474

[B12] Macefield V. G. Fuglevand A. J. Howell J. N. Bigland-Ritchie B. (2000). Discharge behaviour of single motor units during maximal voluntary contractions of a human toe extensor. *J. Physiol.* 528 227–234. 10.1111/j.1469-7793.2000.00227.x 11018121 PMC2270114

[B13] Macefield V. G. Knellwolf T. P. (2018). Functional properties of human muscle spindles. *J. Neurophysiol.* 120 452–467. 10.1152/jn.00071.2018 29668385

[B14] Masuki S. Nose H. (2003). Arterial baroreflex control of muscle blood flow at the onset of voluntary locomotion in mice. *J. Physiol.* 553 191–201. 10.1113/jphysiol.2003.047530 12937292 PMC2343480

[B15] Matsukawa K. Ishii K. Kadowaki A. Liang N. Ishida T. (2012). Differential effect of central command on aortic and carotid sinus baroreceptor-heart rate reflexes at the onset of spontaneous, fictive motor activity. *Am. J. Physiol. Heart Circ. Physiol.* 303 H464–H474. 10.1152/ajpheart.01133.2011 22730386

[B16] Reeder E. J. Green S. (2012). Dynamic response characteristics of hyperaemia in the human calf muscle, effect of exercise intensity and relation to electromyographic activity. *Eur. J. Appl. Physiol.* 112 3997–4013. 10.1007/s00421-012-2362-4 22441829

[B17] Sander M. Macefield V. G. Henderson L. A. (2010). Cortical and brainstem changes in neural activity during static handgrip and post-exercise ischemia in humans. *J. Appl. Physiol.* 108 1691–1170. 10.1152/japplphysiol.91539.2008 20185626

[B18] Simone D. A. Marchettini P. Caputi G. Ochoa J. L. (1994). Identification of muscle afferents subserving sensation of deep pain in humans. *J. Neurophysiol.* 72 883–889. 10.1152/jn.1994.72.2.883 7983543

[B19] Sprangers R. L. Wesseling K. H. Imholz A. L. Imholz B. P. Wieling W. (1991). Initial blood pressure fall on stand up and exercise explained by changes in total peripheral resistance. *J. Appl. Physiol.* 70 523–530. 10.1152/jappl.1991.70.2.523 2022542

[B20] Taylor C. E. Boulton D. Howden E. J. Siebenmann C. Macefield V. G. (2019). Central command increases muscle sympathetic nerve activity more to contracting than non-contracting muscle during rhythmic isotonic leg exercise. *J. Neurophysiol.* 121 1704–1710. 10.1152/jn.00075.2019 30864865

[B21] Tschakovsky M. E. Rogers A. M. Pyke K. E. Saunders N. R. Glenn N. Lee S. J. (2004). Immediate exercise hyperemia in humans is contraction intensity dependent, evidence for rapid vasodilation. *J. Appl. Physiol.* 96 639–644. 10.1152/japplphysiol.00769.2003 14578368

[B22] Victor R. G. Bertocci L. A. Pryor S. L. Nunnally R. L. (1988). Sympathetic nerve discharge is coupled to muscle pH during exercise in humans. *J. Clin. Invest.* 82 1301–1305. 10.1172/jci113730 3170747 PMC442683

[B23] Victor R. G. Pryor S. L. Secher N. H. Mitchell J. H. (1989). Effects of partial neuromuscular blockade on sympathetic nerve responses to static exercise in humans. *Circ. Res.* 65 468–476. 10.1161/01.res.65.2.468 2752552

[B24] Wallin B. G. Burke D. Gandevia S. C. (1992). Coherence between the sympathetic drives to relaxed and contracting muscles of different limbs of human participants. *J. Physiol.* 455 219–233. 10.1113/jphysiol.1992.sp019298 1484355 PMC1175641

